# Posts on central websites need less originality to be noticed

**DOI:** 10.1038/s41598-022-19433-9

**Published:** 2022-09-10

**Authors:** Michele Coscia, Clara Vandeweerdt

**Affiliations:** 1 CS Department, ITU Copenhagen, Rued Langgaards Vej 7, Copenhagen, Denmark; 2 PS Department, Copenhagen University, Øster Farimagsgade 5, Copenhagen, Denmark

**Keywords:** Complex networks, Computer science

## Abstract

Information has major consequences for democracy and society. It is important to understand what factors favor its diffusion. The impact of the content of a message on its likelihood of going viral is poorly understood. Some studies say originality is important for a message not to be overlooked. Others give more relevance to paratextual elements—network centrality, timing, human cognitive limits. Here we propose that originality and centrality interact in a nontrivial way, which might explain why originality by itself is not a good predictor of success. We collected data from Reddit on users sharing hyperlinks. We estimated the originality of each post title and the centrality of the website hosting the shared link. We show that the interaction effect exists: if users share content from a central website, originality no longer increases the odds of receiving at least one upvote. The same is not true for the odds of becoming one of the top 10% scoring posts. We show that originality is concentrated in the domain network: domains in the core of the network produce more original content. Our results imply that research on online information virality needs to take into account the nontrivial interaction between originality and prominence.

## Introduction

The Internet has created information highways through society. Via social media, news feeds, newsgroups and instant messaging, people are able to spread messages with an unprecedented speed and reach. These capabilities have been a force for good—e.g. Wikipedia has improved the potential reach of information—but also for bad—as misinformation campaigns have had severe repercussions on the democratic process^1^ and the effectiveness of vaccination campaigns^2^. Misinformation and fake news are super-spreaders by design^3,4^: if there is no need to be factual, messages can be crafted with virality rather than accuracy in mind. There is a wealth of research showing that misinformation spreads more rapidly^5,6^ and in larger cascades^7^ on social media than true information and has a distinctive style designed to increase arousal^8^—although it should be noted that misinformation on social media is still hotly debated by the scientific community with some studies criticizing these results^9^. In general, it is imperative to understand better how and why messages go viral, to encourage the positive effects of the Web and mitigate its potential negative ones.

One factor that seems to be obviously related to the likelihood of viral diffusion is the content of a message. However, the role of content in determining virality is currently explored mostly qualitatively^10,11^, with a focus on perceived rather than objective characteristics^12^. To objectively quantify the relevant aspects of a message’s content is hard—especially since a significant portion of communication on social media happens via images^13^. Simple quantitative measures of content (such as lexical/syntactic characteristics^14^, number of unique words, or message length^15^) are typically found not to predict success, or to be overwhelmed by other factors.

A few papers, however, explore more sophisticated measures of content and find them to be relevant for message success. Some focus on the sentimental valence of the message—or the amount of arousal it provokes—and connect it with its chances of going viral^16–20^. Other studies look at originality, as research in marketing^21^ and media^22^ suggests that original messages are more likely to catch our collective attention. Image or textual originality indeed seems to help posts be noticed, even if it does not help with success per se^23,24^.

Parallel to the study of successful *content*, research has also looked at the effects of *para-textual* elements on success. For instance, messages are more likely to spread if appreciation from users flows quickly after content publication^15,25,26^. There is also a rich-get-richer effect for messages that obtain an early advantage, caused by the limited attention span of humans^27,28^. One para-textual element that has received particular attention is source centrality: whether the originators of a message^29^ or the websites they use to publish their content^30,31^ sit at the intersection of many communities or are otherwise central in the social structure. Work on the science of success confirms that the *structure* of a network can create a disconnect between the quality of a performance and its success in art^32^, sports^33^, and science^34,35^.

The success-enhancing capacity of network hubs comes partly from their structural ability to reach many other nodes^36^. But hubs also serve as a brand name for the content they host. For instance, a Reddit user may have an easier time getting upvotes for a post with a YouTube video than for one with a video hosted on their personal website. Because of their reach, hubs (such as YouTube) are more likely to be familiar, and the so-called recognition heuristic should drive users to attach more value to their content^37^. At the same time, the recognition heuristic plays a smaller role when other quality cues are available^38,39^—for example, when the content itself is highly original. In the case of social media, most sites display content more prominently than source, meaning that original content may be eye-catching enough for users not to rely on the recognition heuristic.

In this study, we explore the possibility that such interactions between content and para-text are part of the reason why some studies have found surprisingly small effects of content on success. Specifically, we propose that content hosted on websites that are central in the Web, and therefore highly recognizable, need less originality to be receive attention. Put differently, originality has different effects depending on the level of prominence of the information source. Based on existing theory about the recognition heuristic, we formulate three research questions.

As a first research question, we want to know if originality—in conjunction with centrality—predicts *getting noticed*. In other words, we test whether original messages are less likely to be completely overlooked, depending on their source’s centrality. Second, we want to know if originality—in conjunction with centrality—predicts *success*. In other words, we test whether original messages are more likely to be part of the set of most appreciated messages. Finally, we investigate how originality distributes on a network of content sources, to see whether central agents feel the need to be more or less original. The latter question is important because the centrality-originality interaction assumes a different character depending on whether originality is coupled with centrality in the network or if it is completely independent from it.

To answer these questions we focus on posts on Reddit that consist of a web address—which we can place in the Web network allowing us to estimate centrality—and a post title—which allows us to estimate the post’s originality. We focus on titles because they are the first thing users notice, and thus should greatly impact their perception of the content’s overall originality. Further, post titles on Reddit all have comparable formats, where the content of a post can be a text, image, video, or even an audio file—making it challenging to develop a single measure for originality.

## Results

In this section, we show that our first two research questions have opposite answers. On the one hand, if the user shares content originating from a non-central website, originality increases the odds of receiving at least one upvote on Reddit. This is not the case for content from central websites. On the other hand, there is no clear originality-centrality interaction effect when the outcome is post success—measured as being in the top 10% scoring posts. In other words, our results suggest that the originality-centrality interaction is more about getting at least minimal notice than about obtaining actual success.

As for our third research question, we show that both originality and success concentrate in the core of the domain network. Centrality in a website positively correlates with originality, and originality is concentrated in the core of the network. This is counterintuitive: central websites do not need originality as much as peripheral ones, yet their content is more original on average. Results for RQ1 are consistent across different definitions of originality, centrality, success, and also hold true on another social media platform (Twitter). Results for RQ2 are mixed.

### Preliminaries

We use a mixed-effects linear model to investigate the relationship between originality and centrality in defining the success of a post. The following equation specifies our model:

1 $$\begin{aligned} Y_i = \beta _0 + \beta _1 \mathcal {O}_{T_i} + \beta _2 \mathcal {C}_{d_i} + \beta _3 (\mathcal {O}_{T_i} \times \mathcal {C}_{d_i}) + \beta _4 |T_i| + \eta _h + \eta _w + \alpha _s + \epsilon _i, \end{aligned}$$

In this equation:

- $Y_i$ is the success of post *i*. For RQ1, $Y_i$ is the probability of receiving an upvote, $p(>1)$. For RQ2, $Y_i$ is the probability of being in the top 10% scoring posts on Reddit in a day, $p(>10\%)$;
- $T_i$ is the title of post *i* on Reddit, with $|T_i|$ being its length in number of tokens;
- $\mathcal {O}_{T_i}$ is the originality of title $T_i$;
- $d_i$ is the domain of the link shared via that post;
- $\mathcal {C}_{d_i}$ is domain $d_i$’s centrality in the hyperlink network;
- $\eta _h$ and $\eta _w$ are fixed effects for hour of day and day of week in which the post appeared on Reddit;
- $\alpha _s$ is a random effect of the subreddit in which the post was made;
- $\beta _0$ is the intercept and $\epsilon _i$ is the error term.

The focus of this paper is $\beta _3$, which is the coefficient of the interaction between originality and centrality. Existing theory on the recognition heuristic suggests that $\beta _3$ would be negative, as being original would lower the requirement of using a more central (recognizable) website for one’s post. Besides this mixed effects linear model, we also provide results for a fixed effects logit model in the Supplementary Materials Sections 2 and 3.

Hereafter, all coefficients of interest from our regressions are statistically significant with $p < 0.01$—unless otherwise stated. This is due to the size of our dataset. For this reason, rather than focusing on statistical significance, we focus on effect size. The “Methods” section below details how we collected the data, preprocessed the titles $T_i$, estimated a title’s originality $\mathcal {O}_{T_i}$ and a post’s centrality $\mathcal {C}_{d_i}$, and for a justification of our choice of models.

### Originality-centrality interaction: predicting getting noticed (RQ1)

**Figure 1:**
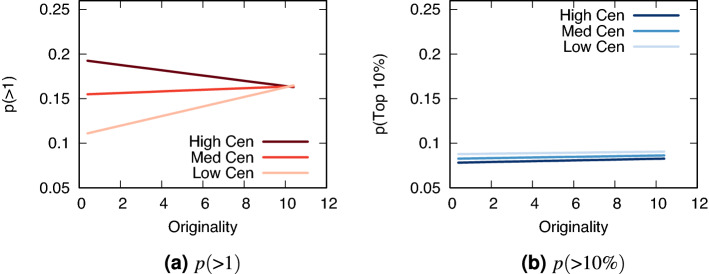
The interaction effect between originality and centrality. Originality on the x axis, centrality (“Cen” in the legend) is the darkness of the line, success on the y axis. (**a**) Probability of getting at least one upvote ($p(>1)$) in red; (**b**) probability of being in the top 10% scoring posts in a day ($p(>10\%)$) in blue.

Figure 1a shows on the y axis the probability of getting one upvote or more. These probabilities are estimated for an “example” post: one made at midnight on Friday in the subreddit r/videos with a post title of length 8—the median title length. We then set website centrality $\mathcal {C}_d$ to high, medium, or low—using the third, second, and first quartile value. Then, we predict $p(>1)$ for growing values of originality $\mathcal {O}_T$. Note that the quantities of interest (namely, the slopes of originality for different levels of centrality) are the same no matter what time, day, subreddit, and post length we choose for the “example” post.

We see that $\beta _3$ from Eq. (1) is negative, which means that as centrality goes up, originality matters less—and vice versa. Being original is actually detrimental if you use a central website: going from the minimum to maximum originality found in the data, the probability of getting an upvote declines from 19.2 to $16.3\%$. Instead, for a non-central website, the probability goes up from 11.1 to $16.5\%$.

Therefore, the answer to our first research question is *yes*: originality interacts with centrality to predict which posts get noticed. The more central the website is, the less originality helps. In other words, the recognition heuristic (triggered by a central website “brand name”) seems to matter somewhat for unoriginal posts, but not for original ones. More importantly, this is true for every way we measure originality, centrality and success—see Supplementary Materials Sections 2 and 3. Supplementary Tables 1–6 report coefficients and significance levels, and show that the basic results hold in a fixed effects logit model.

### Originality-centrality interaction: predicting success (RQ2)

Figure 1b shows that the answer to our second research question is *no*: originality does not interact with centrality to help a post become one of the top 10% performing posts. For high centrality websites, growing originality from its minimum to its maximum value brings the probability from 7.8 to $8.3\%$. For low centrality websites, the probability goes from from 8.8 to $9\%$. None of this is statistically significant. Note that all these probabilities are lower than $10\%$ due to our choices for the fixed effects values.

This analysis suggests that originality matters more for low centrality websites, but only to predict failure—i.e. to increase the chances of getting at least one upvote. Originality has little relevance when it comes to becoming successful—i.e. to be in the top 10% performing posts in a month. Different measurement strategies in the Supplementary Materials Sections 2 and 3, however, do not all confirm this null result. Instead, we see a mix of positive and negative, sometimes statistically significant interaction coefficients (though nearly all are much smaller in size than the corresponding coefficients for RQ1). Therefore, our answer to RQ2 is inconclusive.

### Distribution of originality in the web (RQ3)

A final important factor to understand the relationship between originality and centrality is their correlation in the network of websites. Is it true that posts using more or less central websites achieve significantly different levels of originality? One possible speculation could be that users know that they need additional effort if they want to “make it” when starting from a non-central website. This might explain why in the past the effect of originality on success had been hard to disentangle from centrality.

A simple answer to this question lies in the correlation between centrality and post characteristics. The relationship between centrality and originality appears to be positive and significant, but very small. The Pearson correlation coefficients range from 0.03 to 0.07, depending on the measure of centrality. There is also a positive relationship between the centrality and the average number of upvotes a domain has, with higher coefficients (0.47–0.54).

This analysis is incomplete, though, because networks are multidimensional objects. There are many ways in which the same centrality values—especially the in-degree—could distribute in different structures. For instance, high-in-degree high-original nodes might be scattered in different communities, with the network lacking a core. This would generate a positive degree-originality correlation, but originality would not be concentrated in the network. Supplementary Material Section 4 shows some examples supporting this statement.

To take into account the complex structure of the network, we calculate the network variance^40^ of originality and upvotes, using effective resistance^41^ to estimate the distance between nodes—effective resistance is more robust to random graph fluctuations than the simple shortest path lengths, which can vary greatly with the addition/deletion of a single edge^40^. Network variance is high when original posts are scattered in the periphery, and it is low when they concentrate in the core.

**Figure 2:**
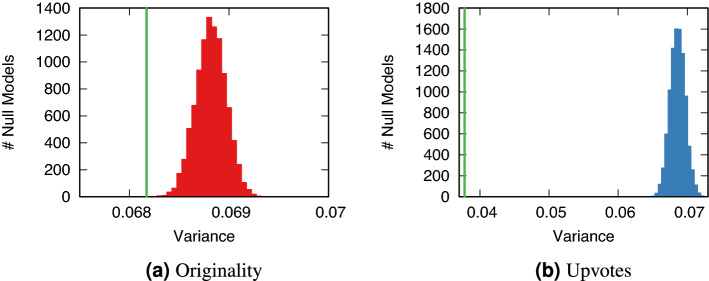
The network variance of originality and upvotes. The observed value of network variance is located at the green vertical bar. The histograms report the value distribution from the null models. The figure shows how many null models (y axis) recorded a given network variance value (x axis) for (**a**) originality $\mathcal {O}_T$ (red), and (**b**) upvotes (blue).

Figure 2a shows the observed value of network variance for originality, compared to the distribution of network variance values of 10, 000 null models. These null models are generated by randomly shuffling the observed originality values across all domains in the network. The actual observed network variance for originality is lower than any of the null models, meaning that originality is more concentrated in the core than we would expect if it were distributed randomly. Figure 2b shows that the same is true for upvotes, where concentration diverges even further from the null models.

In light of this result, the answer to RQ3 is that originality tends to be found in the core of the network, and it positively correlates with centrality. In RQ1 we found that the most central websites benefit the least from originality. Here we find that, in spite of this, these central websites still tend to produce the most original posts. The implication of this finding is that the true role of content originality is harder to disentangle from centrality, because they are not independent from each other—which would explain why it is difficult to study in the literature.

### Robustness checks

To ensure the robustness of our results, we alter all the components of our model one by one with a similar measure and confirm that our results are unaffected by this change—thus they do not hold only for our specific definitions of centrality, originality, success, or even data source. The results of these checks are in Supplementary Materials Sections 2 and 3 and they all confirm the conclusions we have drawn from the previous section: originality interacts with centrality to predict posts getting at least minimal notice (RQ1), but do not predict success (RQ2).

For centrality, we use alternative definitions different from indegree. We estimate a domain $d_i$’s importance via its betweenness and PageRank centrality in the domain network, and via the number of posts sharing content from $d_i$ on Reddit. For originality, we create an alternative measure based on information-theory. It better quantifies the amount of surprising information in a post title, but it is less intuitive. This does not affect the results. We also try altering our definition of success, considering comments rather than upvotes. We then show that our results hold not only for December 2019—the period we consider here—but also for a longer time span (from November 2019 to January 2020). We further show that our results are robust to different ways of cleaning the data, specifically whether we include our analysis domains and subreddits with very few posts—which supposedly provide more noisy data—or whether we choose different thresholds to determine which posts were part of the top performing ones on Reddit. Finally, we use a different data source, repeating our analysis on Twitter. In all cases, our main results hold for RQ1. As noted above, robustness checks lead to mixed results for RQ2.

## Discussion

In this paper we analyze the relationship between the content of a message in social media (specifically its originality), the centrality of the website from which it originates, and its success. According to the literature, content does not seem to play a large role in determining success. In this paper we show why the relationship between the two variables might be hard to properly gauge.

First, originality interacts with centrality in predicting the odds of a message getting noticed at all. Originality matters more when the message originates from a peripheral website. Central platforms can get away with less original messages, most likely because their recognizability helps them get at least minimal attention. Second, the effect of this interaction is only clear when estimating the chances for a message of not failing—on Reddit, this means getting at least one upvote, which represents the minimum possible bar of attention. The effect is unclear when looking at the chances of becoming one of the most successful posts. Finally, originality does not distribute randomly in the network of content creators. Even though central websites could get away with using less originality, on average posts originating from a central website are still more original than posts originating in the periphery. Originality tends to concentrate in the core of the network.

Our paper has a number of limitations, which could be addressed in future work. First, we only investigate social media. While our results hold on two platforms—Reddit and Twitter—it is still unclear whether we discovered something that applies to web communication in general, including for instance personal websites and instant messaging. Second, while our network of content sources contains all the most important nodes in the web network—google.com, facebook.com, twitter.com, etc.—future studies could include a larger portion of the peripheral web.

Finally, we need more confidence about whether we measure originality, centrality and success in the proper way. Our originality measures could be validated by manually annotating a subset of the data. Further, the right measure of prominence for a website might not be in-degree, but average daily pageviews. Future work should also explore success measures more relevant for human society. For instance, success could be measured through high-cost actions such as signing a petition or participating in crowdfunding, rather than simply upvoting or retweeting.

## Methods

We answered our research questions using Reddit data. We downloaded data about all posts sharing a hyperlink on Reddit in December 2019. First, we used a naive Bayes approach to estimate the originality of each post by looking at its title. Next, we created a hyperlink network of the domains of all shared hyperlinks. We then estimated the centrality of the website using network centrality measures on the top-level domain web network.

### Data

#### Reddit

The raw data is 21.2M Reddit posts in the month of December 2019. We are interested in posts sharing a hyperlink: we ignored all “self” posts that are just messages by the user. We also removed all posts self-hosted on Reddit’s image and video hosting. This was done because these posts are not associated with a domain on the Web and thus have no place in our website network. We removed ads and sticky posts—posts that are always on top of the page regardless of the number of upvotes. We did so because these posts are always visible no matter the votes they receive: this would pollute our results. We also removed posts explicitly marked as “Not Safe For Work” (NSFW), normally pornography and gore, as such content might not obey the same rules as regular content. After this step, we were left with 4.8M posts.

We resolved shortened URLs to the main domain they belong to—e.g. flic.kr is transformed into flickr.com. We reduced sub-domains to their parent domain—e.g. i.imgur.com turns into imgur.com. We removed all porn subreddits and webdomains that survived the explicit NSFW filter. We only kept domains that are also found in the Common Crawl data. This reduced the data to 3.8M posts.

We also removed all subreddits with predominant non-English content, like r/brasil or r/norge. This is because our originality score is based on word probabilities, so all words in a language different from the dominant language of the platform (English) would have incorrect originality estimates. Following the same logic, we tokenized all the titles in the dataset by removing English stopwords, stemming the remaining words and keeping only words that are part of the English dictionary—provided by the NLTK Python library—*or* that are sufficiently frequent in the dataset—these include slang words like “wtf” or “subreddit”. If a post had no valid words, it was removed from the dataset.

**Figure 3:**
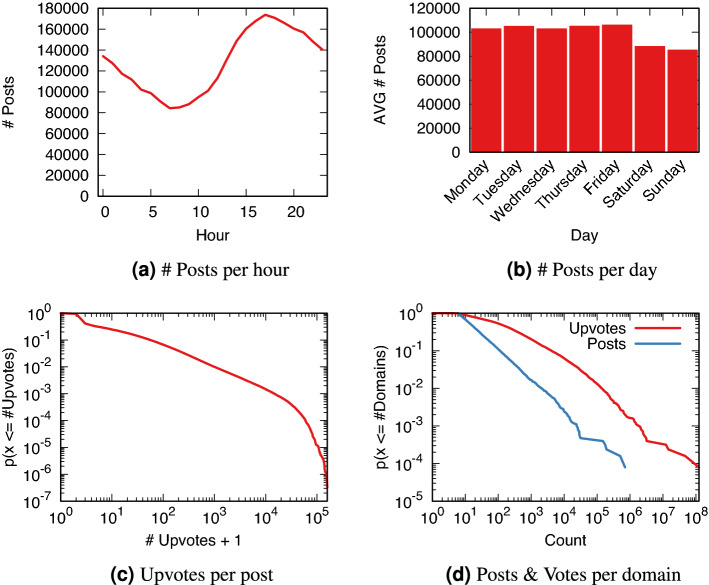
Descriptive statistics of the Reddit dataset. Subfigures (**a**) and (**b**) show raw values, subfigures (**c**) and (**d**) show the complement of the cumulative distribution.

We removed all subreddits with fewer than five surviving posts, and domains that were used less than five times in the data. The final dataset resulted in a collection of 3.1M posts from 11.9k domains and 29.1k subreddits. Figure 3 shows a few summary statistics of the final dataset. Figure 3a,b show trends in the number of posts per hour and per day of the week, which follow the expected patterns of human activity. This is why we controlled for these patterns in our analysis.

The distribution of upvotes per posts and posts and upvotes per domain is skewed (Fig. 3c,d), which means that the majority of posts/domain receive little attention: around 57% of submissions did not receive a single upvote. Most of the attention is grabbed by a few superstar posts/domains. The domain with most posts is youtube.com (694k posts), while the one with most upvotes is imgur.com (114M upvotes).

#### Common crawl

We downloaded the domain graph from the Common Crawl for the November 2019–January 2020 period. Since from the Reddit data we can only reliably obtain top-level domain information, we focus on top-level domains. Nodes in the graph are all of the domains that we found in the Reddit data. Two nodes are connected if there is a hyperlink between any of their webpages.

**Table 1:** Summary statistics of the web graph used.

Measure	Value
# Nodes	11,401
# Edges	2,716,488
Avg Degree	477
Density	0.02
Transitivity	0.32
Avg path length	2.03
Diameter	5

We worked with the largest weakly connected component of the graph. Table 1 reports its summary statistics. The node count is not equal to the domain count in the final Reddit dataset because some of the domains shared on Reddit do not appear in the largest connected component—in fact, they do not have a single hyperlink to the other domains used on Reddit. We needed the website network to have a single connected component in order to perform our robustness checks with betweenness centrality and to address RQ3 with network variance. Thus, posts using those domains were dropped from the analysis.

### Originality

For the purposes of this paper, we decided to use a definition of originality that is as simple and intuitive as possible: an original title is a title that, on average, surprises the reader. In more formal terms, in a surprising title the reader could not predict what word will come next given the word they are reading right now. To the extent that this is a noisy measure of “true” originality, the effects in this paper are lower bound estimates^42^ (p. 320–322). On the other hand, an overly-sophisticated measure might be more difficult to interpret.

We quantified this surprise in terms of probability at a bigram level. A bigram *B* is a sequence of two tokens, $t_1$ and $t_2$. The conditional probability $p(t_2|t_1)$ is the likelihood that a bigram starting with $t_1$ is completed by $t_2$. $p(t_2|t_1)$ is estimated based on posts from the 30 days before *B* was posted (including the day of its posting). If $p(t_3|t_1) < p(t_2|t_1)$, then we say that bigram $(t_1,t_3)$ is more surprising than bigram $(t_1,t_2)$. Bigrams are made up of tokens, not words: there are no stopwords and words are reduced to their semantic roots—this is done to avoid that the originality of a bigram containing “cats” would be rated differently than the same bigram containing “cat” instead. For notation simplicity, if a bigram $B = (t_1,t_2)$, we write $p(t_2|t_1)$ as *p*(*B*).

A title *T* is a sequence of |*T*| bigrams: $$T = \{B_1, B_2, B_3, \ldots , B_{|T|}\}$$. We added two special tokens to every title in the dataset: $$\hat{}$$ to indicate the beginning of the title, and $ to indicate the end. Thus, *T*’s first token ($$B_1$$) is always $$\hat{}$$, and *T*’s last token ($$B_{|T|}$$) is always $. This allows us to estimate the surprise of starting/ending a post title with a given token—a piece of information that would otherwise be ignored.

We could then estimate the average bigram probability of a title *T*:

$$\begin{aligned} \mathcal {T}_T = \dfrac{\sum \nolimits _{i=1}^{|T|} p(B_i)}{|T|}. \end{aligned}$$

We use the notation $\mathcal {T}_T$, because this is a measure of “triviality” of a post—it is higher when the probabilities of each bigram are high. We need to transform it into a measure of originality $\mathcal {O}_T$:

$$\begin{aligned} \mathcal {O}_T = -\log \mathcal {T}_T. \end{aligned}$$

This is a convenient formulation because $\mathcal {T}_T$ is lower than 1—being the average of a set of probabilities—and thus its logarithm is negative. By multiplying the logarithm by $-1$, we obtained a measure that goes from 0 to $\infty$ that is directly proportional to the average level of surprise of the title *T*.

**Figure 4:**
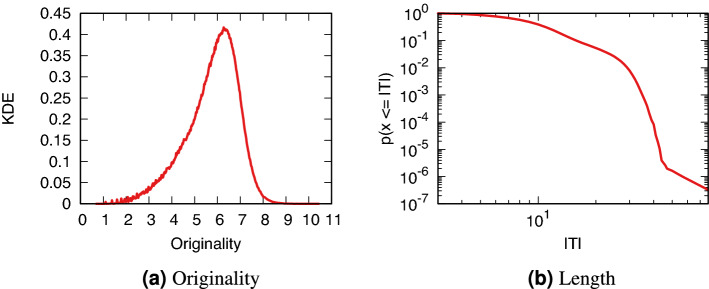
Distributions of $\mathcal {O}_T$ and |*T*|. (**a**) Kernel density estimation (KDE) on the y axis of $\mathcal {O}_T$ values (x axis). (**b**) The probability (y axis) of a post to have a title of length |*T*| (x axis) or shorter.

Figure 4a shows the distribution of the $\mathcal {O}_T$ values across all posts in the Reddit dataset—smoothed via a Kernel Density Estimation (KDE) using a Gaussian kernel with bandwith equal to 0.01. Since $\mathcal {O}_T$ is the result of a logarithm, we can see that the distribution is roughly a lognormal. The distribution is asymmetrical, showing that low $\mathcal {O}_T$ values are more likely than high values, as one would expect.

One objection to our definition of originality could be that it is too linked with the length of a post |*T*|. However, Fig. 4b shows that the distribution of post lengths is different than the distribution of $\mathcal {O}_T$. In fact, their Spearman rank correlation is a mere 0.076. Thus $\mathcal {O}_T$ and |*T*| are capturing different things, and we use |*T*| as a control in our analysis.

The odd tail of the length distribution can be explained by the fact that some subreddits encourage users to post extensive and descriptive titles. For instance, in r/science a user should give enough information in the post title to properly characterize the results of the paper they are linking to (e.g.: https://www.reddit.com/r/science/comments/q39sod/mrna_covid_vaccines_highly_effective_at/, date of last access: October 8th, 2021).

### Centrality

There are many ways to estimate centrality in a network, each capturing a slightly different notion of “importance”. Here we focused on three: in-degree, betweenness centrality^43^, and PageRank^44^. Note that, for betweenness centrality, we used an undirected view of the webgraph, which is preferable as the graph is composed by multiple strongly connected components. The directed betweenness centralities of two nodes in two different strongly connected components would not be comparable. We ignored out-degree centrality because we think it does not meaningfully captures a notion of “importance” in the way it is intended in this paper—it is more a notion of how “active” a node is in creating connections. For the other measures, “importance” means: the ability of capturing hyperlinks (in-degree) from prominent sources (PageRank); or the structural importance in tying together the web graph (betweenness centrality).

The first three columns of Table 2 show the top ranked domains according to these three criteria. The table includes all domains that appear in the top five of at least one centrality measure. From the table we can see that these three network measures all capture something similar, as the set of top ranking nodes is mostly the same in a slightly different order. For this reason, we used the in-degree of domain *d* as our default measure of its centrality, $\mathcal {C}_d$. The other measures were used as robustness checks.

We have an additional measure of importance for web domains that does not use the web graph: number of posts on Reddit that link to the domain. The idea is to capture domains that are “important” simply because they are widely used, whatever the cause for this widespread adoption might be. As the last column of Table 2 shows, this measure captures something rather different from network centrality. Central nodes in the network are used often, but some of the most-used domains are not especially central in the web graph—most prominently, gfycat.com, which is an animated image hosting platform widely used on Reddit. We also provide robustness checks using this alternative measure of importance.

**Table 2:** The top web domains according to their ranks in multiple centrality measures.

Domain	In-degree rank	Betweenness rank	PageRank rank	# Posts rank
twitter.com	1	2	1	3
facebook.com	2	1	3	39
google.com	3	3	2	8
youtube.com	4	5	4	1
instagram.com	5	8	5	25
blogspot.com	11	4	15	7
imgur.com	47	28	34	2
gfycat.com	619	683	450	4
twitch.tv	214	136	73	5

### Models

For our main analyses, we used a mixed-effects regression with an interaction between originality $\mathcal {O}$ and centrality $\mathcal {C}$ and random effects of subreddit. It is specified by Eq. (1).

The model has the benefit that it is easy to interpret: $\beta _3$ is simply the increase in the effect of originality, as centrality goes up by one unit. The effect of originality, in turn, is the increase in probability of a post being noticed or successful, for a one unit increase in originality. However, there are two problems with this model’s assumptions. First, both outcome variables ($p(>1)$ and $p(>10\%)$) are binary. This means the model is a so-called linear probability model. A linear relationship between the predictors and binary outcome might not be plausible in this case, and might lead to predictions of probabilities higher than one or lower than zero. Second, random effects of subreddit might not be reasonable, as they assume that subreddit is not correlated with any of the other predictors.

To address these weaknesses, we checked the robustness of the results with a logit model with subreddit fixed effects, estimated via Conditional Maximum Likelihood Estimation^45,46^ (CMLE). We did not use this fixed-effects logit model as our main model because its coefficients are harder to interpret. However, since the results from two models are compatible—as Supplementary Tables 1–6 show—the interpretations we get from the simpler model are not likely to be misleading.

### Ethics

All data in this study consists of information that Reddit and Twitter users intended to make publicly available. Posts and accounts are not present in our data set if they had been deleted by users before we downloaded the data. We leaned towards analyzing older data, so that Reddit users had the opportunity to delete their content for 1.5 years before it was downloaded. For Twitter, the corresponding period is 4 years. We further filtered out all porn and “self” posts, and deleted full URLs and user IDs, removing all of this potentially more sensitive data from storage immediately after the pre-processing step. In the data set that we made publicly available, we further removed post titles (leaving only their originality score), so that it includes no identifiable information. This research was therefore judged to have minimal risk for participants^47^.

In light of this, no individual-level data has been used for the analysis in this paper and all methods were carried out in accordance with relevant guidelines and regulations for data privacy and ethical concerns. All data used from the analysis is anonymous and open to public access; no permission is needed to access raw data.

## Supplementary Information

Supplementary Information.

## Data Availability

Reddit raw data available at https://files.pushshift.io/reddit/submissions/.Common Crawl raw data available at https://commoncrawl.org/2020/02/host-and-domain-level-web-graphs-novdecjan-2019-2020/.Twitter raw data available at https://files.pushshift.io/twitter/US_PoliticalTweets.tar.gz.Preprocessed data, and scripts for result reproducibility and verifiability, available at https://www.michelecoscia.com/?page_id=2070#scirep22.All datasets are open to public access. No permission is needed to access raw data.
