# Supplementary material for: Posts on central websites need less originality to be noticed
Source: Sci Rep. 2022 Sep 10;12:15265. doi: 10.1038/s41598-022-19433-9 (PMC9464218; doi:10.1038/s41598-022-19433-9)
Supplement: Supplementary file 1 — Supplementary Information. [file 41598_2022_19433_MOESM1_ESM.pdf]

Supplementary Material (Appendix) for: “Posts on Central Websites Need Less Originality to be Noticed”

Michele Coscia[1] **and Clara Vandeweerdt**[2]

[1]CS Dept, ITU Copenhagen, Rued Langgaards Vej 7, Copenhagen, DK, mcos@itu.dk
[2]PS Dept, Copenhagen University, Øster Farimagsgade 5, Copenhagen, DK, clara.vandeweerdt@ifs.ku.dk

ABSTRACT

In this document we show all the robustness checks for the main results of the paper, as well as the full regression tables supporting them.

1 Introduction

This document contains the additional analyses that replicate the results in the main paper for all research questions, using different models and measurement strategies. To summarize the research questions, RQ1 asks: does originality interact with centrality when predicting whether a piece of content will be overlooked? In the paper, we show that the answer is yes: the less central the website from which a piece of content originates, the more original it has to be in order to gather at least one upvote on Reddit. RQ2 asks: does originality interact with centrality when predicting success? In the paper, we show that there is no clear answer: posts at different centrality and originality values do not have significantly different probabilities of being in the top performing posts on Reddit, based on their upvotes. RQ3 asks: is there a correlation in the network between originality and centrality? In the paper, we show that the answer is yes: more central websites tend to produce more original content and originality concentrates in the network. We base this result on our calculation of the network variance of originality.

The results for RQ1 and RQ2 in the main paper are based on regression analysis. In Section 2 we show the values and the significance levels of all relevant coefficients, across all robustness checks we ran. Section 3 goes into detail, explaining what we tested in each robustness check. Section 4 shows some toy examples to aid the intuition of our analysis addressing RQ3. Finally, Section 5 contains the full regression tables for all the robustness checks discussed in Section 3, which contain additional information such as R^2, the number of observations, and more.

2 Overview of Robustness Checks

In this section we examine all the β_3 coefficient values for Equation 1 in the main paper for all robustness checks we performed. The aim is to show that all β_3 coefficients related to RQ1 are negative and significant, thus showing the effect of the interaction between originality and centrality when it comes to gather the first upvote. On the other hand, there is no consistent pattern for the β_3 coefficients related to RQ2, showing that there is no clear relationship between originality and centrality for top-scoring posts.

In all the tables that follow (here and in the next sections), we mark the statistical significance using a classical star notation: * for $p < 0.1$, ** for $p < 0.05$, and *** for $p < 0.01$.

Data	Model	$\mathcal{O}_T$	Success	Periphery	Indegree	Betweenness	PageRank	Posts
12/19	LPM	Main	Upvotes	No	-0.004^{***}	-0.002^{***}	-0.004^{***}	-0.001^{***}
12/19	logit	Main	Upvotes	No	-0.016^{***}	-0.007^{***}	-0.012^{***}	-0.003^{***}
12/19	LPM	Entropy	Upvotes	No	-0.005^{***}	-0.003^{***}	-0.005^{***}	-0.002^{***}
12/19	LPM	Main	Comments	No	-0.002^{***}	-0.001^{***}	-0.002^{***}	0.000
11/19-01/20	logit	Main	Upvotes	No	-0.011^{***}	-0.004^{***}	-0.007^{***}	-0.0004^{***}
12/19	logit	Main	Upvotes	Yes	-0.011^{***}	-0.005^{***}	-0.008^{***}	-0.002^{***}
Twitter	LPM	Main	Retweets	Yes	-0.004^{***}			

Table 1. All β_3 coefficients for all robustness checks we ran for RQ1. *p<0.1; **p<0.05; ***p<0.01

Data	Model	$\mathscr{O}_T$	Success	Periphery	Indegree	Betweenness	PageRank	Posts
12/19	LPM	Main	Upvotes	No	0.0001	0.00004	-0.00005	0.001^{***}
12/19	logit	Main	Upvotes	No	0.002	0.001	-0.0001	0.010^{***}
12/19	LPM	Entropy	Upvotes	No	0.001^{***}	0.0004^{***}	0.001^{***}	0.0003^{***}
12/19	LPM	Main	Comments	No	-0.002^{***}	-0.001^{***}	-0.001^{***}	0.001^{***}
11/19-01/20	logit	Main	Upvotes	No	0.002^{*}	0.001^{*}	0.010^{***}	-0.001
12/19	logit	Main	Upvotes	Yes	0.005^{***}	0.002^{***}	0.003^{**}	0.009^{***}
Twitter	LPM	Main	Retweets	Yes	0.005^{***}			
12/19	LPM	Main	7.5%	No	0.0002	0.0001	0.00005	0.001^{***}
12/19	LPM	Main	12.5%	No	0.0001	0.00002	-0.00001	0.001^{***}

Table 2. All β_3 coefficients for all robustness checks we ran for RQ2. *p<0.1; **p<0.05; ***p<0.01

Table 1 shows all β_3 coefficients for RQ1, as we vary: the definition of centrality (indegree, betweenness, pagerank, or number of posts per domain), the model we run (Linear Probability Model with random effects versus logit with fixed effects), the observation period (December 2019 vs November 2019 - January 2020), the definition of originality (the simple version we use in the paper vs the one based on information entropy), the definition of success (upvotes vs comments), and whether or not we filter underused subreddits and domains. Section 3 contains further details for each of these robustness checks. Note that for analyses with extended data (additional months, subreddits or domains), the Linear Probability Model (LPM) became computationally intractable, meaning we relied on the logit model instead.

We can see that, with one exception, all of these interaction coefficients are negative and significant, confirming our discussion in the main paper. The exception occurs when we use number of posts as a measure of domain centrality and comments as the criterion of success. The explanation for this failure lies in two aspects. First, using number of posts as a ranking is significantly different from all other ranking criteria – as we show in the paper –, since it does not use any network notion of centrality. Second, comments as a criterion for success is much stricter than upvotes: getting one comment is a much more demanding threshold than getting one upvote. Thus in this case RQ1 is stretched, because getting one comment is not the minimum threshold of attention in the same sense as getting one upvote. While this specific finding may be interesting to investigate in future work, it is not especially problematic for our main result.

Table 2 shows all β_3 coefficients for RQ2. The two bottom rows report coefficients for robustness checks specific to RQ2. In these tests we move the threshold of what we consider a "top scoring" post. Rather than using our default threshold of being in the top 10% scoring in terms of upvotes, in these two tests we set the threshold at 7.5% and 12.5%, respectively. Across all tests, the coefficients vary between being statistically non-significant, being positive, and being negative. This means our answer to RQ2 is inconclusive.

3 Robustness Checks Details

3.1 Fixed Effects Logit Model

In this section we discuss the relationship between the simple and intuitive LPM with random effects and the more appropriate but less intuitive logit model with fixed effects. We see that the two models never result in different signs on the interaction coefficients, supporting our decision to use the simple LPM to build an intuition of our results as we do in the main paper.

Concerning RQ1, this view is supported by the first two rows of Table 1, as well as Tables 3 and 4, which contain the results of the regression for the $p(>1)$ variable – the likelihood of getting at least one upvote, i.e. of not failing. Consistently with what we discuss in the main paper, all these coefficients are negative and significant, showing that the more central a platform is, the lower the impact of originality.

The first two rows of Table 2, as well as Tables 5 and 6 contain the results of the regression for the $p(>10\%)$ variable – the likelihood of being in the top 10% scoring posts, i.e. of succeeding. These tables inform our discussion of RQ2 in the main paper. Consistently with what we discuss in the main paper, we do not see a clear negative coefficient for β_3: if anything, we find evidence of a positive coefficient, although this is not robust across all models and centrality definitions.

3.2 Alternative Centrality

The results from the main paper are not dependent on our choice of centrality measure $\mathscr{C}_d$. Here, we replace the indegree with betweenness centrality, PageRank, and number of posts from a specific domain. For RQ1, the sign of β_3 in all these cases is still negative and significant for both the LPM with random effects, and for the CMLE logit model (both with $p < 0.01$) – see the first two rows of Table 1.

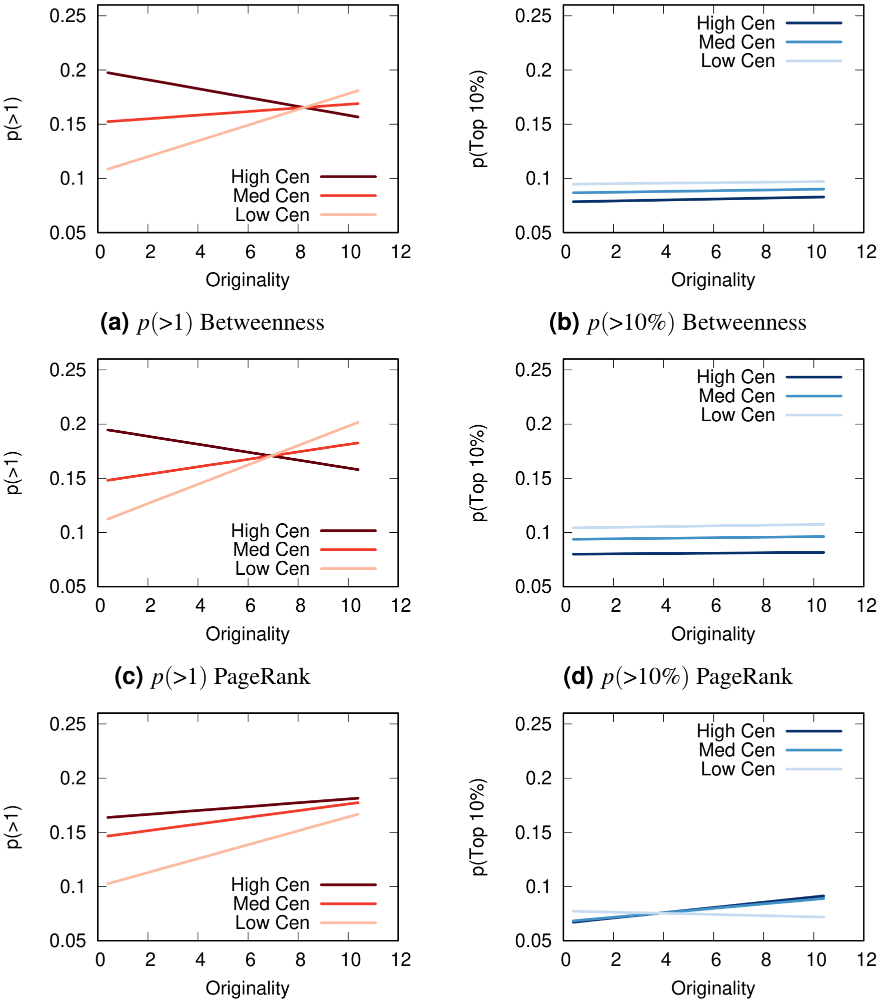

(a) $p(>1)$ Betweenness
(b) $p(>10\%)$ Betweenness
(c) $p(>1)$ PageRank
(d) $p(>10\%)$ PageRank
(e) $p(>1)$ # Posts
(f) $p(>10\%)$ # Posts

Figure 1. The interaction effect between originality and centrality ("Cen" in the legend) for different centrality measures.

Figure 1 is a reproduction of Figure 1 from the main paper, with each row focusing on a different centrality measure. The patterns we observe in all the figures are the same. The difference between high and low centrality platforms is particularly evident when using PageRank as a centrality measure: the probability of getting one upvote shrinks for high centrality sources from 19.5% to 15.8% as originality increases from its minimum to its maximum value, while it increases for low centrality sources from 11.2% to 20.5%. Among these measures, the number of posts has the most different behavior, as it is the least correlated with the other measures – given that it is not a network centrality indicator. However, the effect still holds and it is significant.

With the exception of post counts, the alternative centrality measures also confirm the null result for RQ2 focusing on $p(>10\%)$ – see also the first two rows of Table 2. The coefficients for these alternative measures of centrality are shown in all full regression tables we include in this document for the Reddit dataset, from Table 3 to Table 6.

3.3 Alternative Originality

In our main analyses, we use a relatively simple definition of originality, $\mathcal{O}_T$. Here, we create an originality measure inspired by information entropy[1]. The probability of each bigram B_x $p(B_x)$ is first adjusted by the length of the post: $p^*(B_x) = p(B_x)/(|T|-1)$. Then, we sum over the contributions following Shannon's information entropy as $\sum p^*(B_x)\log_2 p^*(B_x)$. Note that this causes some leftover, because $\sum(p(B_x)/(|T|-1)) < 1$. We use $\lambda_T = 1 - \sum(p(B_x)/(|T|-1))$ to indicate this information leftover and we add it to the sum:

$$IE_T = -\left(\sum(p^*(B_x)\log_2 p^*(B_x)) + (\lambda_T \log_2 \lambda_T)\right).$$

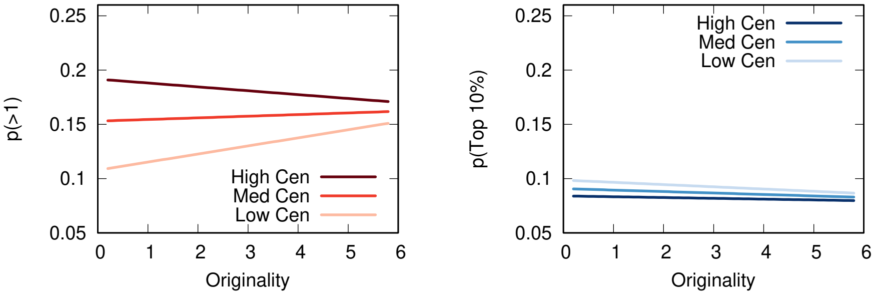

(a) $p(>1)$ **(b)** $p(>10\%)$

Figure 2. The interaction effect between entropy-based originality and centrality ("Cen" in the legend).

If all bigram probabilities $p(B_x)$ are high and roughly equal to each other, the leftover will be small and the information entropy IE_T will be high. IE_T is inversely proportional to originality. We also need to adjust it according to the length of a post, resulting in:

$$\mathscr{O}_T = \log\left(\frac{\log_2 |T|}{IE_T}\right).$$

This measure should capture the originality of a post better than our naive main measure, because it better quantifies the amount of information (i.e. surprise) in a text by using an information-theoretic measure, rather than relying on the most basic framework (Naive Bayes). However, it is less intuitive than our simpler measure.

Results for RQ1 look the same when we use this information-theoretic originality definition. Figure 2 (a) shows the interaction effect of this new $\mathscr{O}_T$ variable with centrality for $p(>1)$. The negative interaction (decreasing originality slope as centrality goes up) is in line with the effect from the original variable we showed in Figure 1 in the main paper. In Figure 2(b), which shows the model for $p(>10\%)$ we instead see a very small but statistically significant positive interaction.

In Tables 7 and 8 – as well as in the third row of Tables 1 and 2 – we see the coefficients for the $p(>1)$ and $p(>10\%)$ regressions when using the alternative $\mathscr{O}_T$ originality definition based on information-theoretic entropy. We confirm the solid negative β_3 coefficient for $p(>1)$, and the small positive coefficients for $p(>10\%)$.

3.4 Alternative Success

Upvotes might not be the right measure of success – an alternative could be that a successful post is one that generates a discussion. In this sense, what matters is not the number of upvotes, but the number of comments. Thus, in this section we change our $p(>1)$ and $p(>10\%)$ variables. Rather than being the probability of getting one upvote and being in the top 10% upvoted posts, they are now the probability of getting at least one comment or being in the top 10% posts in number of comments.

Figure 3 reproduces the results from Figure 1 in the main paper by changing the target variable from upvotes to comments. The result for $p(>1)$ is maintained when switching from an upvote-based definition of success to a comment-based one. For the $p(>1)$ regression, the size of the interaction effect is smaller, but the β_3 coefficient is still negative and significant, confirming the answer to our first research question. Interestingly, the negative interaction now also holds (weakly) for $p(>10\%)$.

A possible explanation as of why the size of the effect is reduced focuses on the rarity of comments over upvotes: receiving one additional comment is a rare occurrence, and it is more similar to being part of the top posts than merely receiving one upvote. In fact, while you need more than 200 upvotes to be in the top 10% scoring posts, you only need 8 comments to rank similarly.

In the fourth row of Tables 1 and 2, and in Tables 9 and 10, we see the coefficients for the $p(>1)$ and $p(>10\%)$ regressions when using comments as the basis of a post's success, rather than upvotes. The coefficients confirm the conclusions above – with the exception of the model using the number of posts as the platform's centrality (as discussed in section 2).

3.5 Consistency Across Months

We obtain the main results for our paper from the December 2019 data on Reddit. As a robustness check, we test whether the results hold for a longer period of time. We replicate the main CMLE logit regression using data from November 2019 to January 2020. Enlarging the time window further introduces too many subreddits and we cannot fit the random effects LPM in the memory we have available – which is also why we run the logit model rather than the LPM as in the other robustness checks. Since we cannot fit this LPM, we keep only December 2019 for the main result and we limit this analysis to a robustness check.

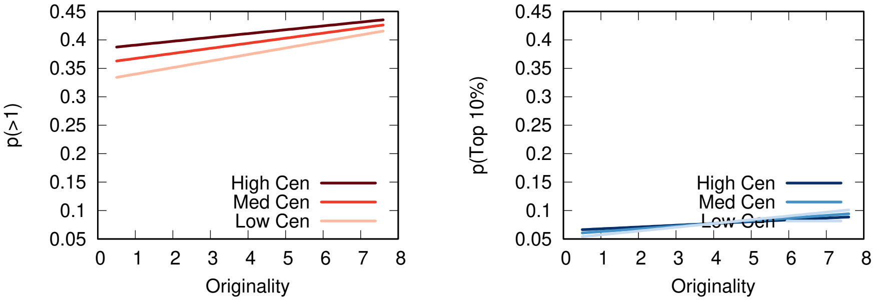

(a) $p(>1)$ **(b)** $p(>10\%)$

Figure 3. The interaction effect between originality and centrality ("Cen" in the legend), in determining a post's success in terms of comments.

The fifth row of Tables 1 and 2, as well as Tables 11 and 12, show the results for RQ1 and RQ2 with this extended dataset. In both cases, we can confirm our answers from the main analysis. The β_3 coefficients are negative and significant for $p(>1)$, confirming the negative centrality-originality interaction for RQ1. For RQ2, we confirm the inconclusiveness of our results.

3.6 Infrequent Domains/Subreddits

One of the cleaning steps we take in the main analysis is removing all domains and subreddits that have not generated at least 5 posts during the observation period. This is done under the assumption that sparsely used domains and subreddits might be dominated by fluctuations and thus add noise to our estimates. However, this has the potential of ignoring the periphery in the website network, and thus we should make sure that we are not biasing our results.

For this reason, in this section we replicate the main LPM regression without excluding the underused domains and subreddits. The sixth row of Tables 1 and 2, along with Tables 13 and 14, report the results. Once again, all results for both RQ1 and RQ2 are consistent with what discussed so far.

3.7 RQ2 Threshold

For RQ2 we want to focus on the most successful posts on Reddit. We thus need a threshold establishing which posts are among the top performers. For the main paper, we decide that the 10% top scoring posts are part of this set. This choice is motivated by the fact that we cannot restrict this set too much, because otherwise the logit regression would not provide accurate results if the outcome variable is mostly zero. On the other hand, we cannot make the set too large, otherwise it would overlap too much with the $p(>1)$ set, making RQ2 indistinguishable from RQ1.

To show that the choice of the 10% threshold is robust, we repeat the experiment answering RQ2 by slightly modifying it. In Tables 15 and 16 – as well as in the bottom section of Table 2 – we change the outcome variable from $p(>10\%)$ to $p(>7.5\%)$ and $p(>12.5\%)$, respectively. In other words, we say that a post is part of the top performing posts if it is in the top 7.5% or 12.5% scoring posts.

The tables show that the erratic behavior of β_3 is confirmed in both cases and thus our negative answer to RQ2 is not dependent on the particular threshold we chose.

3.8 Negative Binomial Model for Upvote Counts

In this paper, we hypothesize (and find) that receiving minimal attention and becoming especially successful are different outcomes with different drivers. Nonetheless, we might be interested in a summary of the effects of originality and centrality on attention (number of upvotes). To do so, we modify our model. Rather than predicting a probability – like $p(>1)$ –, we use the number of upvotes as the outcome variable. The number of upvotes is an over-dispersed count outcome variable – see Figure 3c in the main paper. Therefore, the most appropriate model is a negative binomial regression, which is more flexible than a Poisson model.

Table 17 shows the result. In this case, the interaction effect with all centrality measures – excluding number of posts – is negative. This means that less central websites would fare relatively better, in number of upvotes, with more originality, when compared with highly central websites.

3.9 Alternative Data

Lastly, we address the potential issue that what we are observing might be a Reddit-specific phenomenon. To do so, we collect the US Politics Twitter dataset (`https://files.pushshift.io/twitter/US_PoliticalTweets.tar.gz`, date

of last access: October 19th, 2021). This is a collection of 1.2M tweets sent between August 4th, 2008 and June 6th, 2017. The tweets are all in English from American sources and were collected around a selection of politically-related users. We apply the same data cleaning pipeline we applied to Reddit posts, excluding all the steps related to the web network which we do not have here.

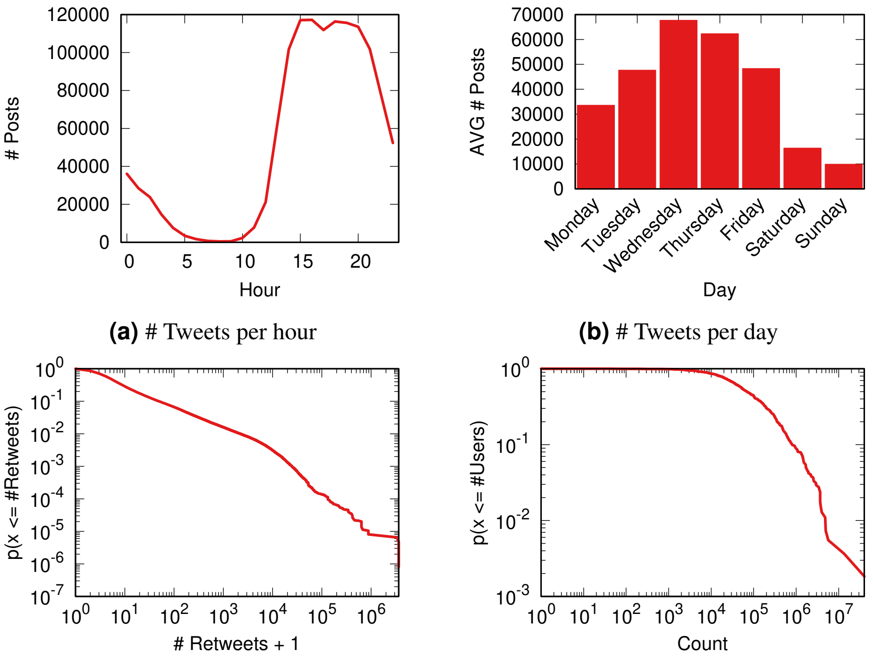

(a) # Tweets per hour **(b)** # Tweets per day

(c) Retweets per post **(d)** Retweets per user

Figure 4. Descriptive statistics for the Twitter dataset.

Figure 4(a-b) shows that Twitter data has comparable hourly and weekly patterns as the Reddit data (Figure 3(b) in the main paper), albeit more extreme. This is due to the fact that the Twitter dataset has fewer users and is concentrated in the US. For this reason, the vast majority of tweets are written when it is daytime in the US. Reddit, on the other hand, has a significant user base outside the US, which continuously adds posts during US nighttime, resulting in a smoother distribution of posts over the day, as we show in the main paper.

In this dataset, we can estimate the originality of the text in the same way we estimate the originality of a Reddit title, since the length is comparable. Figure 5(a) shows that the distribution of originality values in Twitter is similar to the one in Reddit (compare with Figure 4(a) in the main paper). Figure 5(b) allows to compare the tweet length distribution with the Reddit title length distribution we show in Figure 4(b) in the main paper – Twitter lacks the long tail of outliers that Reddit has. Additionally, we report that the median length of a Reddit title is 8 tokens, while for a tweet it is 11.

The success of a tweet is the number of retweets, just as upvotes mark the success of a Reddit post. We use retweets

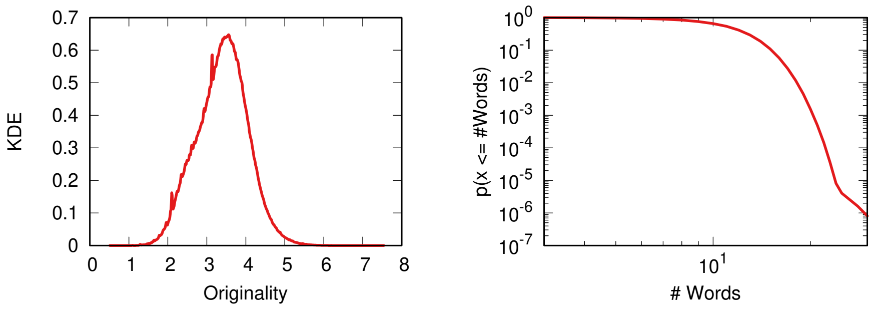

(a) Originality **(b)** Length

Figure 5. Distributions of $\mathscr{O}_T$ and $|T|$ in the Twitter dataset.

because they perform the same visibility-enhancing function on Twitter as upvotes do on Reddit. An upvote makes a post on Reddit float to the top of the front page, which causes more people to see it. Similarly, a retweet publishes the tweet on more Twitter streams, enhancing its visibility. An alternative could be using likes, but crucially likes do not directly enhance a tweet's visibility in an obvious way like retweets do. Thus, we focus on retweets.

Figure 4(c-d) show the distributions of retweets per tweet and per user. A striking difference with the Reddit data is that the odds of a tweet getting retweeted are much higher than the odds of getting one upvote. The probabilities are 86% for the former and 43% for the latter. Compared to Figure 3(c) in the main paper, we see that there is no sharp drop between 0 and 1: the head of the distribution is smoother. The rest of the distribution has a comparable long-tailed shape, although for retweets there is no noticeable exponential cutoff in the tail.

The difference in the probability of getting a retweet vs getting an upvote on Reddit is especially true for high-follower-count users: users with 100k or more followers have a 97% probability of getting at least one retweet per tweet, while users with fewer than 1k followers have a 67% chance. For this reason, as a first outcome variable, we do not look at the probability of getting one or more retweet ($p(>1)$) but rather we look at the probability of getting three or more ($p(>3)$). Overall $p(>1)$ is 43% on Reddit, and $p(>3)$ is 41% in our Twitter sample, rendering the two variables comparable.

As a measure of centrality, we use the in-degree of the author of the tweet – i.e., their follower count. We also have the timestamp of the tweet. Twitter does not have different subreddits – one could use hashtag information, but it is unclear whether it is an appropriate thing to do. In sum, we are able to estimate Equation 1 in the main paper with one slight modification: we leave out the random effect of subreddit α_s.

Figure 6 shows the originality-centrality interaction effect. Our core result is supported: the slope for $p(>3)$ is higher for low-follower count users than it is for high-follower count. The probability of getting three retweets or more – with the specific controls we chose for the example – goes from 22% to 34% for low-follower users, while it decreases from 81% to 80% for high-follower users.

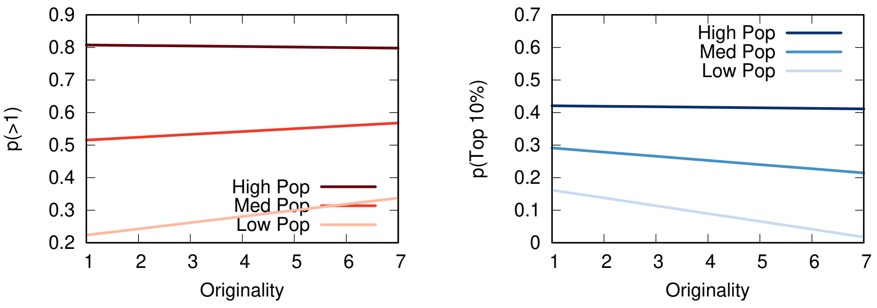

(a) $p(>3)$ **(b)** $p(>10\%)$

Figure 6. The interaction effect between originality and centrality for the Twitter dataset.

Consistently with what we observe on Reddit, the positive answer to our first research question is confirmed – originality matters more for low-centrality users to avoid failure –, and the second answer is still negative. The relationship between originality and centrality for the top 10% scoring posts is unclear: β_3 appears to be positive on Twitter, but it is inconsistently positive, negative or insignificant across all our robustness checks, and thus no real conclusion can be made.

Tables 1 and 2, as well as Tables 18 and 19, show the coefficients for the $p(>3)$ and $p(>10\%)$ regressions in the Twitter dataset. Since in this dataset we only have a single measure of centrality – the follower count –, the tables only have a single column.

4 Network Variance Intuition

In the main paper, we show that the network variance of originality is lower than the one we would expect if originality distributed randomly in the network of websites. We use network variance because the simple correlation of degree and originality is not accurate enough to distinguish different diffusion scenarios. In this section, we provide simple toy examples with two aims. First, we need to support our statement that there are different ways of distributing originality that generate similar correlations with the degree, but that are intuitively different. Second, show the behavior of network variance, to confirm that it corresponds to our intuition.

Consider Figure 7. In Figure 7(a) the high degree highly original nodes are somewhat scattered in the network. On the other hand, in Figure 7(b) they concentrate in the core. We would thus expect to find a difference between these networks: originality is more diffused in Figure 7(a) and more concentrated in Figure 7(b). However, the simple correlation between originality and

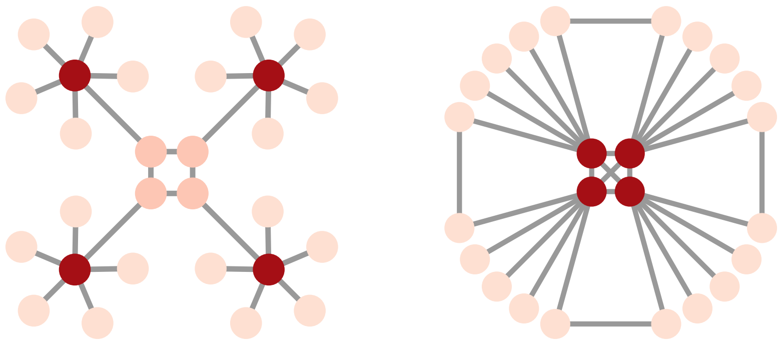

(a) **(b)**

Figure 7. Two toy examples to illustrate the intuition behind network variance. Node color is proportional to the originality of the content the node produces – dark nodes are more original that light nodes.

degree is similar. It is equal to 0.96 and 0.98 in Figures 7(a) and 7(b), respectively. Thus this simple correlation is not sensitive to the differences in these structures. On the other hand, the network variances of originality are 1.29 and 0.43 in Figures 7(a) and 7(b), respectively. This shows that network variance is closer to our intuition when it comes to evaluate how dispersed originality is in the network.

5 Full Regression Tables

In all tables in this section, we report the standard errors of the estimates in parenthesis below the coefficient value. Each row of the top half of the table reports a different β coefficient, while each column reports the coefficient values for a collection of alternative related models. In the bottom half of the table we report information about the fixed effects – for which we omit the coefficients, since they are too many –, as well as statistics about the goodness of fit of the model. We again mark the statistical significance using a classical star notation: * for $p < 0.1$, ** for $p < 0.05$, and *** for $p < 0.01$.

log(posts), log(indegree + 1), log(betwenness), and log(pagerank) are alternative definitions for domain centrality, $\mathscr{C}_d$, with log(indegree + 1) being the main one. Thus, the model of reference is the one in the columns marked as (2). The coefficient of interest is the interaction between centrality and originality (β_3), thus reported in the $\mathscr{O}_T$:* rows, with * being one of the alternative $\mathscr{C}_d$ measurements.

	Dependent variable:			
	$p(>1)$			
	(1)	(2)	(3)	(4)
$\mathscr{O}_T$	0.015^{***}	0.033^{***}	-0.010^{***}	-0.022^{***}
	(0.001)	(0.001)	(0.0004)	(0.001)
log(posts + 1)	0.013^{***}			
	(0.0004)			
log(indegree + 1)		0.040^{***}		
		(0.001)		
log(betwenness)			0.016^{***}	
			(0.0002)	
log(pagerank)				0.027^{***}
				(0.001)
$\|T\|$	0.003^{***}	0.003^{***}	0.003^{***}	0.003^{***}
	(0.0001)	(0.0001)	(0.0001)	(0.0001)
$\mathscr{O}_T$:log(posts + 1)	-0.001^{***}			
	(0.0001)			
$\mathscr{O}_T$:log(indegree + 1)		-0.004^{***}		
		(0.0001)		
$\mathscr{O}_T$:log(betwenness)			-0.002^{***}	
			(0.00004)	
$\mathscr{O}_T$:log(pagerank)				-0.004^{***}
				(0.0001)
Constant	0.442^{***}	0.292^{***}	0.699^{***}	0.765^{***}
	(0.004)	(0.005)	(0.003)	(0.005)
Day FE	Yes	Yes	Yes	Yes
Hour FE	Yes	Yes	Yes	Yes
Subreddit FE	No	No	No	No
Observations	3,073,791	3,073,791	3,073,791	3,073,791
Log Likelihood	-1,254,038.000	-1,250,163.000	-1,253,780.000	-1,256,524.000
Akaike Inf. Crit.	2,508,148.000	2,500,398.000	2,507,631.000	2,513,120.000
Bayesian Inf. Crit.	2,508,614.000	2,500,864.000	2,508,097.000	2,513,586.000
Note:				*p<0.1; **p<0.05; ***p<0.01

Table 3. The LPM with subreddit random effects for $p(>1)$.

	Dependent variable:			
	$p(>1)$			
	(1)	(2)	(3)	(4)
$\mathscr{O}_T$	0.045***	0.137***	−0.034***	−0.068***
	(0.003)	(0.004)	(0.002)	(0.003)
log(posts + 1)	0.032***			
	(0.002)			
log(indegree + 1)		0.132***		
		(0.003)		
log(betwenness)			0.051***	
			(0.001)	
log(pagerank)				0.079***
				(0.003)
$\|T\|$	0.008***	0.008***	0.008***	0.008***
	(0.0002)	(0.0002)	(0.0002)	(0.0002)
$\mathscr{O}_T$:log(posts + 1)	−0.003***			
	(0.0003)			
$\mathscr{O}_T$:log(indegree + 1)		−0.016***		
		(0.0005)		
$\mathscr{O}_T$:log(betwenness)			−0.007***	
			(0.0002)	
$\mathscr{O}_T$:log(pagerank)				−0.012***
				(0.0005)
Day FE	Yes	Yes	Yes	Yes
Hour FE	Yes	Yes	Yes	Yes
Subreddit FE	Yes	Yes	Yes	Yes
Observations	3,073,791	3,073,791	3,073,791	3,073,791
R^2	0.001	0.002	0.002	0.001
Max. Possible R^2	0.992	0.992	0.992	0.992
Log Likelihood	-7,505,901.000	-7,504,466.000	-7,505,464.000	-7,506,289.000
Wald Test	3,856.160***	6,418.090***	4,605.500***	3,086.750***
LR Test	3,869.606***	6,740.764***	4,744.906***	3,094.612***
Score (Logrank) Test	3,857.350***	6,428.579***	4,605.293***	3,086.800***
Note:				*p<0.1; **p<0.05; ***p<0.01

Table 4. The CMLE logit model with subreddit fixed effects for $p(>1)$.

	Dependent variable:			
	$p(>10\%)$			
	(1)	(2)	(3)	(4)
$\mathscr{O}_T$	-0.008^{***}	-0.0003	0.001	-0.0001
	(0.001)	(0.001)	(0.0005)	(0.001)
log(posts + 1)	-0.003^{***}			
	(0.0004)			
log(indegree + 1)		-0.005^{***}		
		(0.001)		
log(betwenness)			-0.003^{***}	
			(0.0004)	
log(pagerank)				-0.008^{***}
				(0.001)
$\lvert T \rvert$	0.002^{***}	0.002^{***}	0.002^{***}	0.002^{***}
	(0.0001)	(0.0001)	(0.0001)	(0.0001)
$\mathscr{O}_T$:log(posts + 1)	0.001^{***}			
	(0.0001)			
$\mathscr{O}_T$:log(indegree + 1)		0.0001		
		(0.0001)		
$\mathscr{O}_T$:log(betwenness)			0.00004	
			(0.0001)	
$\mathscr{O}_T$:log(pagerank)				-0.00005
				(0.0001)
Constant	0.072^{***}	0.076^{***}	0.023^{***}	-0.008
	(0.005)	(0.007)	(0.003)	(0.006)
Day FE	Yes	Yes	Yes	Yes
Hour FE	Yes	Yes	Yes	Yes
Subreddit FE	No	No	No	No
Observations	1,272,639	1,272,639	1,272,639	1,272,639
Log Likelihood	-164,669.800	-164,667.600	-164,368.500	-163,865.400
Akaike Inf. Crit.	329,411.600	329,407.200	328,808.900	327,802.800
Bayesian Inf. Crit.	329,845.600	329,841.200	329,243.000	328,236.900
Note:				*p<0.1; **p<0.05; ***p<0.01

Table 5. The LPM with subreddit random effects for $p(>10\%)$.

	Dependent variable:			
	$p(>10\%)$			
	(1)	(2)	(3)	(4)
$\mathscr{O}_T$	-0.101^{***}	-0.015	0.005	-0.001
	(0.010)	(0.015)	(0.006)	(0.012)
log(posts + 1)	-0.044^{***}			
	(0.006)			
log(indegree + 1)		-0.058^{***}		
		(0.011)		
log(betwenness)			-0.031^{***}	
			(0.005)	
log(pagerank)				-0.075^{***}
				(0.010)
$\lvert T \rvert$	0.023^{***}	0.024^{***}	0.024^{***}	0.024^{***}
	(0.001)	(0.001)	(0.001)	(0.001)
$\mathscr{O}_T$:log(posts + 1)	0.010^{***}			
	(0.001)			
$\mathscr{O}_T$:log(indegree + 1)		0.002		
		(0.002)		
$\mathscr{O}_T$:log(betwenness)			0.001	
			(0.001)	
$\mathscr{O}_T$:log(pagerank)				-0.0001
				(0.002)
Day FE	Yes	Yes	Yes	Yes
Hour FE	Yes	Yes	Yes	Yes
Subreddit FE	Yes	Yes	Yes	Yes
Observations	1,272,639	1,272,639	1,272,639	1,272,639
R^2	0.003	0.003	0.003	0.004
Max. Possible R^2	0.694	0.694	0.694	0.694
Log Likelihood	-751,054.600	-750,965.500	-750,781.300	-750,461.500
Wald Test	$3,893.860^{***}$	$4,091.030^{***}$	$4,476.850^{***}$	$5,112.140^{***}$
LR Test	$3,763.497^{***}$	$3,941.747^{***}$	$4,310.168^{***}$	$4,949.815^{***}$
Score (Logrank) Test	$3,913.634^{***}$	$4,109.962^{***}$	$4,494.975^{***}$	$5,132.549^{***}$
Note:				*p<0.1; **p<0.05; ***p<0.01

Table 6. The CMLE logit model with subreddit fixed effects for $p(>10\%)$.

	Dependent variable:			
	$p(>1)$			
	(1)	(2)	(3)	(4)
$\mathscr{O}_T$	0.031***	0.044***	−0.012***	−0.027***
	(0.001)	(0.001)	(0.0004)	(0.001)
log(posts + 1)	0.018***			
	(0.0003)			
log(indegree + 1)		0.039***		
		(0.0005)		
log(betwenness)			0.016***	
			(0.0002)	
log(pagerank)				0.026***
				(0.001)
$\|T\|$	0.003***	0.003***	0.003***	0.003***
	(0.0001)	(0.0001)	(0.0001)	(0.0001)
$\mathscr{O}_T$:log(posts + 1)	−0.002***			
	(0.0001)			
$\mathscr{O}_T$:log(indegree + 1)		−0.005***		
		(0.0001)		
$\mathscr{O}_T$:log(betwenness)			−0.003***	
			(0.00005)	
$\mathscr{O}_T$:log(pagerank)				−0.005***
				(0.0001)
Constant	0.395***	0.296***	0.698***	0.759***
	(0.004)	(0.004)	(0.003)	(0.004)
Day FE	Yes	Yes	Yes	Yes
Hour FE	Yes	Yes	Yes	Yes
Subreddit FE	No	No	No	No
Observations	3,073,791	3,073,791	3,073,791	3,073,791
Log Likelihood	-1,253,496.000	-1,249,815.000	-1,253,393.000	-1,256,276.000
Akaike Inf. Crit.	2,507,065.000	2,499,702.000	2,506,858.000	2,512,623.000
Bayesian Inf. Crit.	2,507,531.000	2,500,168.000	2,507,324.000	2,513,089.000
Note:				*p<0.1; **p<0.05; ***p<0.01

Table 7. The LPM for $p(>1)$ (entropy-based $\mathscr{O}_T$).

	Dependent variable:			
	$p(>10\%)$			
	(1)	(2)	(3)	(4)
$\mathcal{O}_T$	-0.005^{***}	-0.006^{***}	0.001	0.004^{***}
	(0.001)	(0.001)	(0.001)	(0.001)
log(posts + 1)	0.0003			
	(0.0004)			
log(indegree + 1)		-0.007^{***}		
		(0.001)		
log(betwenness)			-0.004^{***}	
			(0.0003)	
log(pagerank)				-0.012^{***}
				(0.001)
$\|T\|$	0.002^{***}	0.002^{***}	0.002^{***}	0.002^{***}
	(0.0001)	(0.0001)	(0.0001)	(0.0001)
$\mathcal{O}_T$:log(posts + 1)	0.0003^{***}			
	(0.0001)			
$\mathcal{O}_T$:log(indegree + 1)		0.001^{***}		
		(0.0002)		
$\mathcal{O}_T$:log(betwenness)			0.0004^{***}	
			(0.0001)	
$\mathcal{O}_T$:log(pagerank)				0.001^{***}
				(0.0002)
Constant	0.045^{***}	0.102^{***}	0.022^{***}	-0.027^{***}
	(0.005)	(0.006)	(0.003)	(0.005)
Day FE	Yes	Yes	Yes	Yes
Hour FE	Yes	Yes	Yes	Yes
Subreddit FE	No	No	No	No
Observations	1,272,639	1,272,639	1,272,639	1,272,639
Log Likelihood	-164,713.600	-164,646.700	-164,339.200	-163,831.500
Akaike Inf. Crit.	329,499.300	329,365.400	328,750.400	327,735.000
Bayesian Inf. Crit.	329,933.300	329,799.400	329,184.400	328,169.000
Note:				*p<0.1; **p<0.05; ***p<0.01

Table 8. The LPM for $p(>10\%)$ (entropy-based $\mathcal{O}_T$).

	Dependent variable:			
	$p(>1)$			
	(1)	(2)	(3)	(4)
$\mathscr{O}_T$	0.011***	0.027***	0.003***	-0.002^{**}
	(0.001)	(0.001)	(0.0003)	(0.001)
log(posts + 1)	0.005***			
	(0.0003)			
log(indegree + 1)		0.026***		
		(0.0005)		
log(betwenness)			0.010***	
			(0.0002)	
log(pagerank)				0.015***
				(0.001)
$\|T\|$	0.003***	0.003***	0.003***	0.003***
	(0.00005)	(0.00005)	(0.00005)	(0.00005)
$\mathscr{O}_T$:log(posts + 1)	0.00004			
	(0.0001)			
$\mathscr{O}_T$:log(indegree + 1)		-0.002^{***}		
		(0.0001)		
$\mathscr{O}_T$:log(betwenness)			-0.001^{***}	
			(0.00004)	
$\mathscr{O}_T$:log(pagerank)				-0.002^{***}
				(0.0001)
Constant	0.298***	0.166***	0.431***	0.457***
	(0.004)	(0.004)	(0.003)	(0.005)
Day FE	Yes	Yes	Yes	Yes
Hour FE	Yes	Yes	Yes	Yes
Subreddit FE	No	No	No	No
Observations	3,073,791	3,073,791	3,073,791	3,073,791
Log Likelihood	-1,065,534.000	-1,062,958.000	-1,065,527.000	-1,067,209.000
Akaike Inf. Crit.	2,131,140.000	2,125,988.000	2,131,126.000	2,134,489.000
Bayesian Inf. Crit.	2,131,606.000	2,126,454.000	2,131,592.000	2,134,955.000
Note:				*p<0.1; **p<0.05; ***p<0.01

Table 9. The LPM for $p(>1)$ (comments version).

	Dependent variable:			
	$p(>10\%)$			
	(1)	(2)	(3)	(4)
$\mathscr{O}_T$	-0.002^{*}	0.018^{***}	0.001^{*}	-0.002^{*}
	(0.001)	(0.002)	(0.001)	(0.001)
log(posts + 1)	-0.006^{***}			
	(0.001)			
log(indegree + 1)		0.007^{***}		
		(0.001)		
log(betwenness)			0.001^{*}	
			(0.001)	
log(pagerank)				-0.004^{***}
				(0.001)
$\|T\|$	0.006^{***}	0.006^{***}	0.006^{***}	0.006^{***}
	(0.0001)	(0.0001)	(0.0001)	(0.0001)
$\mathscr{O}_T$:log(posts + 1)	0.001^{***}			
	(0.0001)			
$\mathscr{O}_T$:log(indegree + 1)		-0.002^{***}		
		(0.0002)		
$\mathscr{O}_T$:log(betwenness)			-0.001^{***}	
			(0.0001)	
$\mathscr{O}_T$:log(pagerank)				-0.001^{***}
				(0.0002)
Constant	0.149^{***}	0.031^{***}	0.087^{***}	0.055^{***}
	(0.008)	(0.010)	(0.005)	(0.008)
Day FE	Yes	Yes	Yes	Yes
Hour FE	Yes	Yes	Yes	Yes
Subreddit FE	No	No	No	No
Observations	1,191,817	1,191,817	1,191,817	1,191,817
Log Likelihood	-557,847.100	-557,879.200	-557,567.800	-556,882.800
Akaike Inf. Crit.	1,115,766.000	1,115,830.000	1,115,208.000	1,113,838.000
Bayesian Inf. Crit.	1,116,198.000	1,116,262.000	1,115,639.000	1,114,269.000
Note:				*p<0.1; **p<0.05; ***p<0.01

Table 10. The LPM for $p(>10\%)$ (comments version).

	Dependent variable:			
	$p(>1)$			
	(1)	(2)	(3)	(4)
$\mathscr{O}_T$	0.013***	0.090***	−0.019***	−0.037***
	(0.002)	(0.002)	(0.001)	(0.002)
log(posts + 1)	0.013***			
	(0.001)			
log(indegree + 1)		0.090***		
		(0.002)		
log(betwenness)			0.032***	
			(0.001)	
log(pagerank)				0.043***
				(0.002)
$\|T\|$	0.008***	0.007***	0.008***	0.008***
	(0.0001)	(0.0001)	(0.0001)	(0.0001)
$\mathscr{O}_T$:log(posts + 1)	−0.0004***			
	(0.0001)			
$\mathscr{O}_T$:log(indegree + 1)		−0.011***		
		(0.0003)		
$\mathscr{O}_T$:log(betwenness)			−0.004***	
			(0.0001)	
$\mathscr{O}_T$:log(pagerank)				−0.007***
				(0.0003)
Day FE	Yes	Yes	Yes	Yes
Hour FE	Yes	Yes	Yes	Yes
Subreddit FE	Yes	Yes	Yes	Yes
Observations	9,306,230	9,306,230	9,306,230	9,306,230
R^2	0.001	0.002	0.001	0.001
Max. Possible R^2	0.997	0.997	0.997	0.997
Log Likelihood	-27,253,158.000	-27,250,614.000	-27,252,888.000	-27,254,193.000
Wald Test	9,075.040***	13,771.800***	9,493.400***	7,012.570***
LR Test	9,048.475***	14,135.550***	9,587.129***	6,977.291***
Score (Logrank) Test	9,076.766***	13,780.460***	9,493.061***	7,013.519***
Note:				*p<0.1; **p<0.05; ***p<0.01

Table 11. The CMLE logit model with subreddit random effects for $p(>1)$ for the November 2019 - January 2020 period.

	Dependent variable:			
	p(>10%)			
	(1)	(2)	(3)	(4)
$\mathcal{O}_T$	-0.115^{***} (0.006)	-0.019^{**} (0.008)	0.001 (0.003)	-0.009 (0.007)
log(posts + 1)	-0.045^{***} (0.003)			
log(indegree + 1)		-0.053^{***} (0.006)		
log(betwenness)			-0.029^{***} (0.003)	
log(pagerank)				-0.068^{***} (0.006)
$\|T\|$	0.023^{***} (0.0004)	0.023^{***} (0.0004)	0.023^{***} (0.0004)	0.024^{***} (0.0004)
$\mathcal{O}_T$:log(posts + 1)	0.010^{***} (0.001)			
$\mathcal{O}_T$:log(indegree + 1)		0.002^{*} (0.001)		
$\mathcal{O}_T$:log(betwenness)			0.001^{*} (0.0004)	
$\mathcal{O}_T$:log(pagerank)				-0.001 (0.001)
Day FE	Yes	Yes	Yes	Yes
Hour FE	Yes	Yes	Yes	Yes
Subreddit FE	Yes	Yes	Yes	Yes
Observations	3,908,207	3,908,207	3,908,207	3,908,207
R^2	0.003	0.003	0.003	0.004
Max. Possible R^2	0.755	0.755	0.755	0.755
Log Likelihood	-2,739,405.000	-2,739,345.000	-2,738,900.000	-2,737,882.000
Wald Test	$12{,}351.650^{***}$	$12{,}530.770^{***}$	$13{,}459.040^{***}$	$15{,}493.160^{***}$
LR Test	$11{,}941.570^{***}$	$12{,}062.610^{***}$	$12{,}952.640^{***}$	$14{,}987.120^{***}$
Score (Logrank) Test	$12{,}415.680^{***}$	$12{,}591.710^{***}$	$13{,}517.800^{***}$	$15{,}557.270^{***}$
Note:				*p<0.1; **p<0.05; ***p<0.01

Table 12. The CMLE logit model with subreddit random effects for $p(> 10\%)$ for the November 2019 - January 2020 period.

	Dependent variable:			
	$p(>1)$			
	(1)	(2)	(3)	(4)
$\mathscr{O}_T$	0.037***	0.106***	−0.024***	−0.046***
	(0.003)	(0.003)	(0.001)	(0.003)
log(posts + 1)	0.029***			
	(0.001)			
log(indegree + 1)		0.091***		
		(0.002)		
log(betwenness)			0.039***	
			(0.001)	
log(pagerank)				0.057***
				(0.002)
$\lvert T \rvert$	0.008***	0.008***	0.008***	0.008***
	(0.0002)	(0.0002)	(0.0002)	(0.0002)
$\mathscr{O}_T$:log(posts + 1)	−0.002***			
	(0.0002)			
$\mathscr{O}_T$:log(indegree + 1)		−0.011***		
		(0.0004)		
$\mathscr{O}_T$:log(betwenness)			−0.005***	
			(0.0002)	
$\mathscr{O}_T$:log(pagerank)				−0.008***
				(0.0003)
Day FE	Yes	Yes	Yes	Yes
Hour FE	Yes	Yes	Yes	Yes
Subreddit FE	Yes	Yes	Yes	Yes
Observations	3,265,350	3,265,350	3,265,350	3,265,350
R^2	0.001	0.002	0.002	0.001
Max. Possible R^2	0.992	0.992	0.992	0.992
Log Likelihood	-7,786,740.000	-7,785,842.000	-7,786,669.000	-7,787,374.000
Wald Test	4,755.270***	6,368.010***	4,819.210***	3,500.860***
LR Test	4,780.625***	6,575.755***	4,922.131***	3,512.507***
Score (Logrank) Test	4,757.213***	6,374.743***	4,820.080***	3,501.428***
Note:				*p<0.1; **p<0.05; ***p<0.01

Table 13. The CMLE logit model with subreddit fixed effects for $p(>1)$ keeping infrequent domains and subreddits.

	Dependent variable:			
	p(>10%)			
	(1)	(2)	(3)	(4)
$\mathscr{O}_T$	-0.102^{***}	-0.044^{***}	0.015^{***}	0.020^{**}
	(0.009)	(0.013)	(0.006)	(0.010)
log(posts + 1)	-0.035^{***}			
	(0.005)			
log(indegree + 1)		-0.055^{***}		
		(0.009)		
log(betwenness)			-0.030^{***}	
			(0.004)	
log(pagerank)				-0.066^{***}
				(0.008)
$\|T\|$	0.023^{***}	0.023^{***}	0.024^{***}	0.024^{***}
	(0.001)	(0.001)	(0.001)	(0.001)
$\mathscr{O}_T$:log(posts + 1)	0.009^{***}			
	(0.001)			
$\mathscr{O}_T$:log(indegree + 1)		0.005^{***}		
		(0.001)		
$\mathscr{O}_T$:log(betwenness)			0.002^{***}	
			(0.001)	
$\mathscr{O}_T$:log(pagerank)				0.003^{**}
				(0.001)
Day FE	Yes	Yes	Yes	Yes
Hour FE	Yes	Yes	Yes	Yes
Subreddit FE	Yes	Yes	Yes	Yes
Observations	1,372,271	1,372,271	1,372,271	1,372,271
R^2	0.003	0.003	0.003	0.003
Max. Possible R^2	0.689	0.689	0.689	0.689
Log Likelihood	-800,158.600	-800,308.500	-800,223.600	-799,955.100
Wald Test	$4,304.950^{***}$	$4,024.770^{***}$	$4,200.700^{***}$	$4,741.860^{***}$
LR Test	$4,183.797^{***}$	$3,884.011^{***}$	$4,053.648^{***}$	$4,590.793^{***}$
Score (Logrank) Test	$4,324.551^{***}$	$4,042.808^{***}$	$4,218.379^{***}$	$4,760.306^{***}$
Note:				*p<0.1; **p<0.05; ***p<0.01

Table 14. The CMLE logit model with subreddit fixed effects for $p(> 10\%)$ keeping infrequent domains and subreddits.

	Dependent variable:			
	$p(>7.5\%)$			
	(1)	(2)	(3)	(4)
$\mathscr{O}_T$	-0.007^{***}	-0.001	0.0004	0.0003
	(0.001)	(0.001)	(0.0004)	(0.001)
log(posts + 1)	-0.003^{***}			
	(0.0004)			
log(indegree + 1)		-0.004^{***}		
		(0.001)		
log(betwenness)			-0.003^{***}	
			(0.0003)	
log(pagerank)				-0.007^{***}
				(0.001)
$\|T\|$	0.002^{***}	0.002^{***}	0.002^{***}	0.002^{***}
	(0.0001)	(0.0001)	(0.0001)	(0.0001)
$\mathscr{O}_T$:log(posts + 1)	0.001^{***}			
	(0.0001)			
$\mathscr{O}_T$:log(indegree + 1)		0.0002		
		(0.0001)		
$\mathscr{O}_T$:log(betwenness)			0.0001	
			(0.0001)	
$\mathscr{O}_T$:log(pagerank)				0.00005
				(0.0001)
Constant	0.053^{***}	0.060^{***}	0.011^{***}	-0.016^{***}
	(0.005)	(0.006)	(0.003)	(0.005)
Day FE	Yes	Yes	Yes	Yes
Hour FE	Yes	Yes	Yes	Yes
Subreddit FE	No	No	No	No
Observations	1,272,639	1,272,639	1,272,639	1,272,639
Log Likelihood	-18,063.370	-18,077.360	-17,836.860	-17,423.970
Akaike Inf. Crit.	36,198.740	36,226.720	35,745.720	34,919.940
Bayesian Inf. Crit.	36,632.780	36,660.760	36,179.760	35,353.970
Note:				*p<0.1; **p<0.05; ***p<0.01

Table 15. The LPM with subreddit random effects for $p(> 7.5\%)$.

	Dependent variable:			
	p(>12.5%)			
	(1)	(2)	(3)	(4)
$\mathcal{O}_T$	-0.010^{***}	-0.0001	0.001	-0.0001
	(0.001)	(0.001)	(0.001)	(0.001)
log(posts + 1)	-0.004^{***}			
	(0.0005)			
log(indegree + 1)		-0.005^{***}		
		(0.001)		
log(betwenness)			-0.003^{***}	
			(0.0004)	
log(pagerank)				-0.008^{***}
				(0.001)
$\|T\|$	0.003^{***}	0.003^{***}	0.003^{***}	0.003^{***}
	(0.0001)	(0.0001)	(0.0001)	(0.0001)
$\mathcal{O}_T$:log(posts + 1)	0.001^{***}			
	(0.0001)			
$\mathcal{O}_T$:log(indegree + 1)		0.0001		
		(0.0002)		
$\mathcal{O}_T$:log(betwenness)			0.00002	
			(0.0001)	
$\mathcal{O}_T$:log(pagerank)				-0.0001
				(0.0001)
Constant	0.096^{***}	0.097^{***}	0.039^{***}	0.004
	(0.006)	(0.008)	(0.004)	(0.006)
Day FE	Yes	Yes	Yes	Yes
Hour FE	Yes	Yes	Yes	Yes
Subreddit FE	No	No	No	No
Observations	1,272,639	1,272,639	1,272,639	1,272,639
Log Likelihood	-274,550.600	-274,605.000	-274,264.200	-273,689.100
Akaike Inf. Crit.	549,173.200	549,281.900	548,600.400	547,450.200
Bayesian Inf. Crit.	549,607.300	549,716.000	549,034.400	547,884.200
Note:				*p<0.1; **p<0.05; ***p<0.01

Table 16. The LPM with subreddit random effects for $p(> 12.5\%)$.

Dependent Variable:	upvotes			
Model:	(1)	(2)	(3)	(4)
Variables				
originality	-0.0639***	0.1149***	-0.0757***	-0.1293***
	(0.0238)	(0.0174)	(0.0158)	(0.0266)
log(posts+1)	0.0060			
	(0.0150)			
length	0.0240***	0.0233***	0.0236***	0.0241***
	(0.0039)	(0.0039)	(0.0039)	(0.0039)
originality × log(posts+1)	0.0045*			
	(0.0023)			
log(indegree+1)		0.1407***		
		(0.0203)		
originality × log(indegree+1)		-0.0178***		
		(0.0028)		
log(betwenness)			0.0458***	
			(0.0088)	
originality × log(betwenness)			-0.0084***	
			(0.0014)	
log(pagerank)				0.0394*
				(0.0205)
originality × log(pagerank)				-0.0164***
				(0.0032)
Fixed-effects				
hour	Yes	Yes	Yes	Yes
day	Yes	Yes	Yes	Yes
subreddit	Yes	Yes	Yes	Yes
Fit statistics				
Observations	3,073,699	3,073,699	3,073,699	3,073,699
Squared Correlation	0.03343	0.03330	0.03340	0.03377
Pseudo R^2	0.15603	0.15593	0.15584	0.15604
BIC	20,429,757.4	20,432,121.6	20,434,166.3	20,429,484.7
Over-dispersion	0.45626	0.45598	0.45573	0.45632

Clustered (subreddit) standard-errors in parentheses
*Signif. Codes: ***: 0.01, **: 0.05, *: 0.1*

Table 17. The negative binomial model for total upvote count.

	Dependent variable:
	$p(>3)$
$\mathscr{O}_T$	0.050***
	(0.005)
log(followers + 1)	0.131***
	(0.002)
$\|T\|$	0.011***
	(0.0001)
$\mathscr{O}_T$:log(followers + 1)	−0.004***
	(0.001)
Constant	−0.819***
	(0.018)
Day FE	Yes
Hour FE	Yes
Subreddit RE	No
Observations	1,235,138
R^2	0.100
Adjusted R^2	0.100
Residual Std. Error	0.466
F Statistic	4,166.490***
Note:	*p<0.1; **p<0.05; ***p<0.01

Table 18. The LPM for $p(>3)$ in the Twitter dataset.

	Dependent variable:
	$p(>10\%)$
$\mathscr{O}_T$	-0.061^{***}
	(0.004)
log(followers + 1)	0.051^{***}
	(0.001)
$\|T\|$	0.005^{***}
	(0.0001)
$\mathscr{O}_T$:log(followers + 1)	0.005^{***}
	(0.0004)
Constant	-0.453^{***}
	(0.013)
Day FE	Yes
Hour FE	Yes
Subreddit FE	No
Observations	735,785
R^2	0.113
Adjusted R^2	0.113
Residual Std. Error	0.282
F Statistic	$2,838.874^{***}$
Note:	*p<0.1; **p<0.05; ***p<0.01

Table 19. The LPM for $p(>10\%)$ in the Twitter dataset.

References

1. Shannon, C. E. A mathematical theory of communication. *The Bell system technical journal* **27**, 379–423 (1948).
